# Thyroid Eye Disease

**DOI:** 10.3390/life12122084

**Published:** 2022-12-12

**Authors:** Ramy Rashad, Raquel Pinto, Emily Li, Mahsa Sohrab, Alberto G. Distefano

**Affiliations:** 1 Department of Ophthalmology, Boston University School of Medicine, Boston, MA 02118, USA; 2 Department of Ophthalmology, Wilmer Eye Institute, Johns Hopkins School of Medicine, Baltimore, MD 21205, USA; 3 Independent Researcher, Greenwich, CT 06831, USA; 4 Department of Ophthalmology, Icahn School of Medicine at Mount Sinai, New York Eye and Ear Infirmary, New York, NY 10003, USA

**Keywords:** thyroid eye disease, thyroid-associated orbitopathy, Grave’s disease, Hashimoto’s thyroiditis, chronic lymphocytic thyroiditis, euthyroid eye disease, orbital inflammation, exophthalmos, proptosis, teprotumumab

## Abstract

Thyroid eye disease (TED), an autoimmune inflammatory disorder of the orbit, presents with a potential array of clinical sequelae. The pathophysiology behind TED has been partially characterized in the literature. There remain certain elusive mechanisms welcoming of research advances. Disease presentation can vary, but those that follow a characteristic course start mild and increase in severity before plateauing into an inactive phase. Diagnosis and evaluation include careful physical examination, targeted laboratory work up, appropriate imaging studies, and tailored treatment regimens. Special consideration may apply to certain populations, such as pediatric and pregnant patients.

## 1. Introduction

Thyroid eye disease (TED) is an inflammatory disease of orbital tissue characterized by infiltration of lymphocytic cells, orbital fat expansion, and extraocular muscle swelling [1]. The gravity of thyroid eye disease lies in its sight-threatening, debilitating, and disfiguring potential. Despite extensive ongoing research about TED, the disorder remains elusive in its exact pathophysiology, prevention, and ideal treatment. The following discussion will present an evidence-based overview of what is currently known and clinically relevant to facilitate appropriate understanding, recognition, work up, monitoring, and management of TED. Clinicians can use this up-to-date and comprehensive review to guide treatment of patients with thyroid eye disease. Additionally, this discussion sheds light on recent advances in our understanding and treatment of the disease, a highly dynamic landscape with several ongoing promising clinical trials. As thyroid eye disease can profoundly affect quality of life, such a review is especially helpful and imperative for providing clinicians and patients with the most comprehensive and up-to-date care.

## 2. Background

### 2.1. Epidemiology

The overall prevalence of TED is approximately 0.25%, though 25–50% of Grave’s disease patients develop clinically evident TED [2,3]. Risk factors include demographic (female sex and European ethnicity), anatomical (wider lateral wall orbital angle), and environmental (tobacco smoking, radioactive iodine therapy) considerations [2,4,5]. It occurs six times more commonly among females than males, at rates of 16 per 100,000 and 2.9 per 100,000, respectively. Although this disparity reflects the higher incidence of hyperthyroidism among females, severe TED occurs four times more commonly in males. Smoking increases the risk of TED by seven to eight times [2]. It also confers a greater likelihood of disease progression. Given the influence of smoking on TED, it confounds epidemiologic studies that have investigated incidence and severity among different ethnic populations. Nevertheless, research suggests Europeans have a lower risk of developing TED than Asians [4,5]. The risk of disease development or progression after radioactive iodine (RAI) therapy is 15–39%, which is greater than those of antithyroid medications (3–21%) and surgery (15%) [4]. Smoking and RAI are additive risk factors for developing TED [4,5].

Although there is no known distinct genetic susceptibility to TED, epigenetic factors and polymorphisms may be at play. Studies have suggested an association with polymorphisms in genes encoding human leukocyte antigen, cytotoxic T-lymphocyte antigen 4, interleukin 23 receptor, protein tyrosine thyroglobulin, and thyroid-stimulating hormone receptor [2]. Quality studies are warranted to establish clear genetic associations with the development and severity of TED.

### 2.2. Pathophysiology

The pathophysiology behind TED is multifaceted. Disease activity is driven by orbital inflammation, with immune cell infiltration leading to activation and proliferation of myofibroblasts and adipocytes [1,6]. Production of glycosaminoglycans (hyaluronic acid and chondroitin sulfate), chemokines, and cytokines augment expansion of orbital soft-tissue volume and inflammatory activity [1].

The targeted effector in TED orbital inflammation is activated orbital fibroblasts. They express thyroid-stimulating hormone receptors (TSHR), activated by autoantibodies, anti-TSHR, and anti-insulin-like growth factor-1 (IGF-1); all of which are expressed at higher levels in Graves’ TED patients [2]. In turn, activated fibroblasts increase adipogenesis and production of extracellular matrix components, hyaluronic acid, and proinflammatory molecules [2,6]. Fibroblasts may be more susceptible to activation in environments with high oxidative stress, such as with tobacco smoking [1].

Activation of TSHR and IGF-1 receptors also stimulate secretion of inflammatory cytokines and infiltration of immunocompetent cells into the orbit [6]. This leads to increased production of interferon-γ, interleukin (IL)-1, IL-6, IL-8, macrophage chemoattractant protein, transforming growth factor-β, and thyrotropin receptor antibodies (TSAb) [2,6]. Levels of TSAb have been shown to correlate with TED activity and severity [1]. Additionally, there is perivascular and diffuse infiltration of CD4+ and CD8+ T cells, B lymphocytes, plasma cells, macrophages, and mast cells [2,6].

Cases of TED in which anti-TSHR antibodies are absent, such as in Hashimoto’s thyroiditis and euthyroid TED, exhibit autoantibodies against ocular muscle and orbital soft-tissue antigens: calsequestrin, collagen XIII, flavoprotein, and protein G2s [1,6,7].

Orbital inflammation leads to extraocular muscle edema, which can restrict motility and cause diplopia. Increased orbital fat and immune infiltration increase the volume of orbital soft tissue, leading to venous congestion and proptosis. Worsening congestion can compress the optic nerve, leading to neuropathy and permanent vision loss [1]. Elevated orbital pressure can lead to forward protrusion of the eye, known as exophthalmos. 

## 3. Clinical Evaluation

Ocular and systemic disease present simultaneously in 40% of TED patients. Of those who develop TED, 85–90% are hyperthyroid, 5–6% are euthyroid, and 4% are hypothyroid at disease onset [2,5,8]. Typical presenting symptoms include dry eyes, red eyes, double vision, and pain with eye movement. The most common sign is eyelid retraction, followed by exophthalmos and eye motility limitation; additional findings include periorbital swelling and conjunctival injection. In severe cases, patients may present with vision loss secondary to ocular surface compromise and/or optic neuropathy [2]. 

Several grading scales are available to document TED severity and track inflammatory activity. The European Group on Graves’ Orbitopathy (EUGOGO) protocol and the VISION classification (V: vision, optic neuropathy; I: inflammation, congestion; S: strabismus, motility restriction; A: appearance, exposure) provide clinical grading. The NOSPECS (N: no signs or symptoms; O: only signs; S: soft-tissue involvement; P: proptosis; E: extraocular muscle involvement; C: corneal involvement; S: sight loss due to optic nerve compression) classification and Mourits system are useful for research purposes [2].

### 3.1. Mild Disease

**Figure 1:**
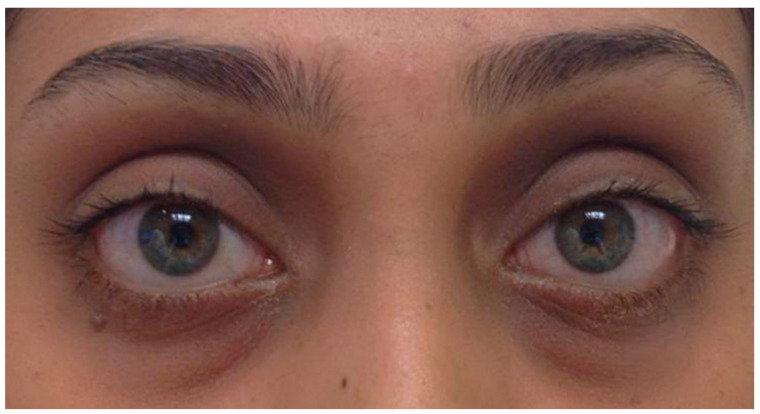
Mild Thyroid Eye Disease. External color photograph showing mild signs of bilateral lower eyelid retraction, lateral upper eyelid flare, periorbital edema, and trace conjunctival injection.

TED may present with minimal findings that can easily go unrecognized or be misdiagnosed. Patients may report foreign body sensation, photophobia, or tearing, which are indicative of dry eyes secondary to exposure keratopathy (corneal disease) [8]. Examination may reveal mild periorbital swelling, proptosis <3 mm over the normal value for race and gender, eyelid retraction <2 mm, and conjunctival injection (Figure 1) [1,8].

### 3.2. Moderate Disease

**Figure 2:**
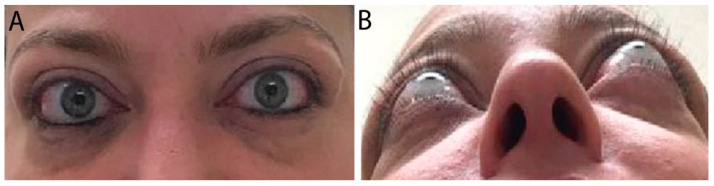
External color photograph of a patient with chronic moderate thyroid eye disease with (**A**) upper and lower eyelid retraction, lateral flare of the upper eyelids, and moderate periorbital fat protrusion. (**B**) Proptosis, best seen in “worm’s eye view” (chin-up position).

In moderate TED, patients have more apparent signs and symptoms. Orbital inflammation and congestion can lead to significant periorbital edema, conjunctival swelling and injection, corneal disease, pain with eye movements, diplopia, proptosis >2 mm, and eye motility restriction (Figure 2) [1,8].

### 3.3. Severe Disease

**Figure 3:**
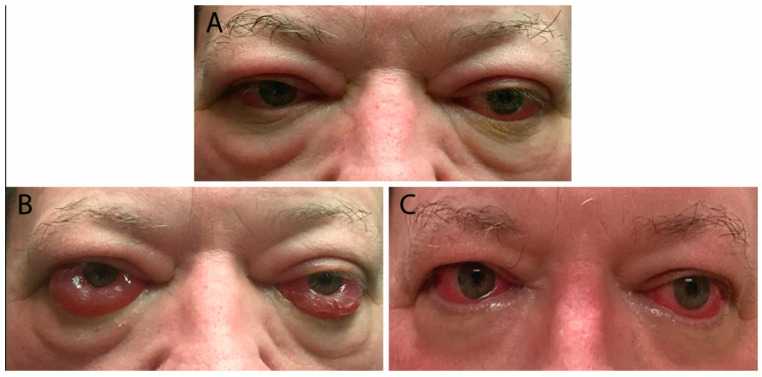
(**A**) External color photograph of patient with acute moderate thyroid eye disease beginning two months after radioactive iodine therapy. (**B**) One month later with progression to severe thyroid eye disease. Exam shows increased conjunctival injection and chemosis. Sixty milligrams of oral prednisone with adjunctive radiotherapy was started. (**C**) Significant improvement in conjunctival and periorbital edema after one month. Steroids were slowly tapered over eight months.

Risk factors for severe TED include advanced age, male gender, and smoking [5]. Patients presenting with optic neuropathy and/or corneal breakdown require urgent referral to ophthalmology as these are definite indications for surgical decompression. Red flag symptoms include persistently blurred or decreased vision, scotomas, and decreased color vision. Examination may reveal lagophthalmos (incomplete eyelid closure), absent Bell’s phenomenon (upward movement of the eye during attempted eye closure which protects the cornea from exposure), relative afferent pupillary defect (if one eye is affected more than the other), corneal opacity, and decreased vision (Figure 3) [8]. Patients with optic neuropathy may not display dramatic proptosis, as orbital soft-tissue volume expansion within an enclosed area is more likely to lead to compression than when there is forward movement of the globe to provide more retrobulbar space [8,9].

### 3.4. Natural Course

Although the natural course of TED can vary, disease typically follows an archetype known as Rundle’s curve. TED begins with an active inflammatory phase that rapidly progresses to maximally severe disease before improving to a stable, inactive plateau [2,5,8]. Onset of orbitopathy tends to coincide with development of Graves’ hyperthyroidism, occurring within 18 months of each other in 60–85% of patients [8]. The self-limited disease course is unique compared to other autoimmune disorders and may be ascribed to the absence of lymphoid tissue in the orbit [2]. There may be slow improvement after months to years of inactive, plateaued disease. Nevertheless, most patients with moderate-to-severe TED do not achieve complete recovery back to normal, with proptosis being the least likely manifestation to resolve [8].

## 4. Diagnosis

### 4.1. Differential

TED can present in innumerable ways, warranting careful clinical evaluation and targeted ancillary testing based on each individual patient’s constellation of signs and symptoms. The most common cause of both unilateral and bilateral proptosis in adults is TED [10,11]. When patients present with proptosis, the differential also includes primary and secondary orbital tumors, carotid-cavernous fistula, unilateral myopia, orbital pseudotumor, orbital myositis, and systemic inflammatory disease [8,11]. Binocular diplopia carries a long list of differential diagnoses, the most relevant of which are myasthenia gravis (characteristically variable diplopia associated with ptosis), orbital tumors, orbital myositis, and orbital pseudotumor in patients with TED. Upper eyelid retraction can develop in midbrain neurological disease, and lower eyelid retraction can occur along with decreased ocular motility in chronic progressive extranuclear ophthalmoplegia [8]. Restrictive limitations in extraocular motility are associated with orbital fracture, Brown syndrome, Duane syndrome, and congenital fibrosis of the extraocular muscles [12]. When TED is suspected, complete work up encompasses multiple investigatory modalities. Diagnosis is based on ophthalmic signs and symptoms on physical examination, presence of thyroid autoimmunity on laboratory testing, and exclusion of an alternative diagnosis [1].

### 4.2. Laboratory Testing

Serologic work up should assess thyroid function and thyroid autoimmunity. Thyroid testing includes thyroid-stimulating hormone (TSH) and free T4; if both are normal but there is a strong suspicion for TED, free T3 can be considered. Antibody testing must look for antithyroid peroxidase (TPO), antithyroid-stimulating immunoglobulins (TSI), TSHR-stimulating antibodies (TSAb), and thyroid-binding inhibitory immunoglobulins (TBIIs) [1,2]. TSI levels correlate to TED inflammation and can serve as a marker for disease activity and prognosis. Free T3 and TPO levels have also been correlated with active inflammation [1].

### 4.3. Imaging

Orbital imaging is a helpful component of TED evaluation, as 90% of patients with Graves’ disease have abnormalities in imaging studies [13]. Methods to visualize and monitor disease activity in TED include orbital ultrasound, computed tomography (CT), magnetic resonance imaging (MRI), somatostatin receptor scintigraphy with radio-labeled somatostatin analog (Octreotide, octreoscan), and optical coherence tomography (OCT) [11].

#### 4.3.1. Orbital Ultrasound

Ultrasound is a cost-effective screening modality that can detect extraocular muscle enlargement, though is less sensitive than CT and MRI. There are two types: the A-scan can identify enlargement greater than the 95th percentile; and the B-scan visualizes TED signs such as posterior scalloped orbital fat secondary to muscle enlargement, widening of the echo-free area between orbital fat and bone, and optic nerve sheath “doubling” in optic neuropathy. Advantages of orbital ultrasound include no patient exposure to radiation, short investigation time, and accessibility in ophthalmology clinics. Nevertheless, orbital ultrasound is rarely used to detect and monitor TED given the availability and improved accuracy of CT and MRI [11].

#### 4.3.2. Computed Tomography

**Figure 4:**
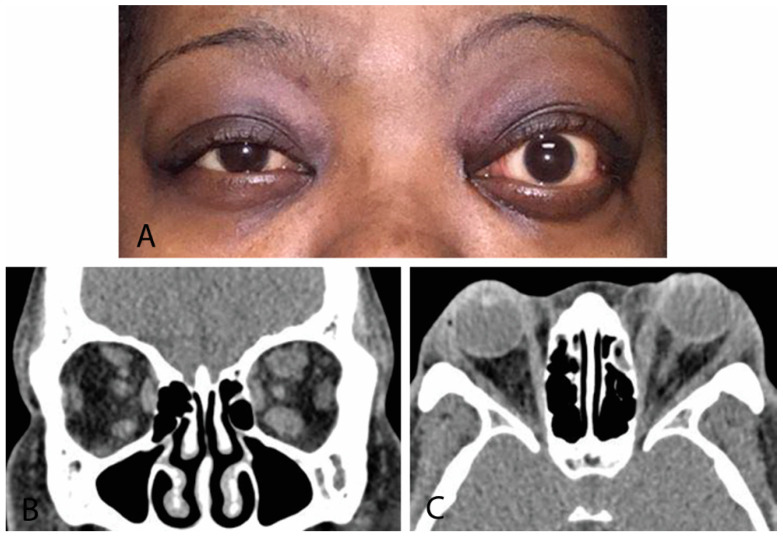
(**A**) External color photograph of chronic moderate thyroid eye disease with asymmetric proptosis on left more than right. (**B**) Coronal CT of orbits with thickening of recti muscles on left more than right. (**C**) Sagittal cut of same CT scan.

CT provides superior bone resolution and is essential to orbital surgeons when planning decompression surgery [2,11,13]. Additionally, it is readily available with short study duration, accurately assesses volume of orbital tissues, and provides precise imaging of the orbital apex and sinuses. Disadvantages include patient radiation exposure and inability to monitor disease activity. Radiographic signs suggestive of TED include extraocular muscle enlargement sparing tendon insertions, slight bowing of the medial orbital wall (“Coca-Cola sign”), optic nerve compression by enlarged extraocular muscles, fat prolapse with optic nerve compression, and absence of orbital masses, vascular enlargement, and sinus involvement (Figure 4). The inferior rectus is affected most commonly, and rarely, the lacrimal gland may be enlarged [11]. Orbital CT is required for preoperative assessment for orbital decompression surgery [13].

#### 4.3.3. Magnetic Resonance Imaging

**Figure 5:**
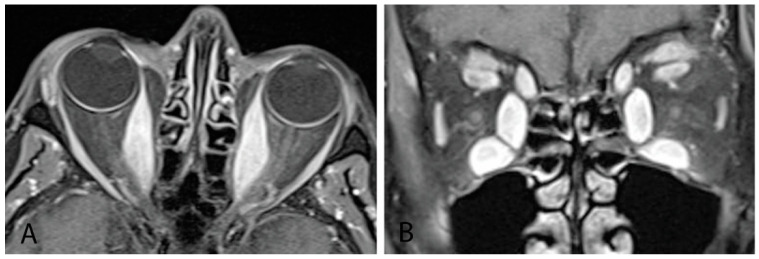
(**A**) MRI orbits with gadolinium with sagittal cut showing thickening of bilateral medial recti muscles. (**B**) Coronal cut from same patient showing thickening of all bilateral inferior, medial, and superior recti muscles, as well as levator palpebrae superioris and superior oblique muscles.

MRI with gadolinium contrast and fat suppression is the modality of choice for identifying and monitoring active disease [2,11]. It provides high-resolution images that enable orbital soft-tissue differentiation and the ability to detect changes in water content (interstitial edema) within extraocular muscles and orbital fat expansion without radiation exposure (Figure 5). MRI should be used to assess whether vision loss in TED is secondary to optic nerve compression or a noncompressive etiology and to differentiate inflammatory from fibrotic tissue [11,13]. Serial orbital MRI investigations can be used to monitor response to treatment and activity of orbital disease [13]. Barriers to MRI include relatively high cost, greater length of study time, and potential claustrophobia. It is less helpful than CT for bony structure evaluation [11].

#### 4.3.4. Octreoscan

Octreoscan, also known as somatostatin receptor scintigraphy, uses octreotide, a radio-labeled somatostatin analog, to detect inflammation through accumulation associated with lymphocytes, myoblasts, fibroblasts, endothelial cells, or localized blood pooling from inflammation-induced venous stasis. It is highly sensitive for detecting inflammation of orbital tissue, and it can be used to predict response to immunosuppressive therapy and monitor disease activity in TED. However, it poses significant disadvantages, including whole-body radiation exposure, lack of morphologic assessment, and significant cost. Octreoscan is mainly reserved for cases in which disease activity is unclear from clinical evaluation and MRI is not available [11].

#### 4.3.5. Optical Coherence Tomography (OCT)

OCT is a noninvasive imaging technique routinely used in ophthalmology, particularly in glaucoma and neuro-ophthalmic settings. Recently, changes in the retinal nerve fiber and ganglion cell layers have been studied in TED. Subclinical thinning has been noted, which can be important for disease monitoring and treatment [14]. Optical coherence tomography angiography (OCT-A) has also been used to study peripapillary and macular vessel density changes, which can further provide subclinical information on disease progression [15]. Lastly, subfoveal choroidal thickness has been correlated with thyroid eye disease activity and is currently under further review for its potential role in disease prognostication [16]. It is important to note that the above OCT changes are not specific to thyroid eye disease and may be confounded by other ocular diseases.

### 4.4. Biopsy

Most cases of TED in patients with unknown thyroid dysfunction can be diagnosed without surgical biopsy. However, suspicion of malignancy serves as a potential indication for extraocular muscle or orbital soft-tissue biopsy [8]. The main histologic features of TED include normal extraocular muscle fibers separated by abnormally wide spaces of connective tissue and hydrophilic extracellular matrix materials, T lymphocytes, and macrophages. There may be focal accumulations of B lymphocytes and natural killer cells. After chronic compression, muscle fibers display a fibrotic, atrophic appearance [9].

## 5. Additional Considerations

### 5.1. Pediatric Thyroid Eye Disease

#### 5.1.1. Epidemiology

Although the risk of developing TED in children is comparable to that of adults, disease severity is characteristically mild and regresses after restoring euthyroidism [1,17]. Pediatric TED typically presents in Graves’ disease but may develop in Hashimoto’s thyroiditis. The overall incidence is 0.79–6.5 per 100,000 children, occurring at a rate of 1.7–3.5 per 100,000 per year. TED in children is more common among girls than boys and occurs more frequently in the adolescent population (11–18 years old; 68.2%) than in children younger than 11 years old (31.8%). The latter trend may be related to increased smoking among adolescents. Even passive smoking appears to impose risk, as evidenced by a higher incidence of TED in children younger than 11 years old in countries where tobacco use is higher [1].

#### 5.1.2. Clinical Considerations

Pediatric TED usually manifests with signs and symptoms similar to those of adult TED but is milder and tends to regress after systemic treatment. The most common symptoms in children are pain, foreign body sensation, photophobia, and excessive tearing. Diplopia is sometimes reported. The most common physical examination findings include upper eyelid lag in downgaze (Graefe sign), upper eyelid retraction, and proptosis. With puberty, rates of more serious consequences, such as extraocular muscle restriction and ocular surface compromise secondary to exposure, occur [1]. Studies that have investigated TED disease activity after RAI, drug therapy, or subtotal thyroidectomy in children found improvement in 90%, 73%, and 75%, respectively [17]. More severe complications, such as eye motility restriction, tend to improve more often than proptosis, which may be attributed to the normal increase in exophthalmometry (measurement of eye protrusion) with age [1].

Long-term sequelae of pediatric TED include an association with refractive changes and a potential for permanent poor vision. TED increases the risk of developing myopia and astigmatism in children. Additionally, patients who are already near-sighted experience higher rates of myopic progression [1]. Amblyopia can develop if the visual pathway does not fully develop during childhood. Causes in TED include sensory deprivation from ocular surface compromise, uncorrected changes in refractive error, and restrictive strabismus.

#### 5.1.3. Management

Clinicians can use the same physical examination, laboratory markers, and disease classification systems to evaluate TED activity and severity in children [1].

### 5.2. Pregnancy

While it is well established that the systemic manifestations of Graves’ hyperthyroidism improve during pregnancy and rebound in the postpartum period, there remains a dearth of rigorous evaluation of TED activity during pregnancy and in the postpartum period [10,18]. If TED is linked to systemic autoimmunity, the natural course of TED should reflect the same improvement and subsequent rebound activity. However, conclusions from one retrospective study suggest this is not the case. TED activity varied considerably in seven patients with Graves’ hyperthyroidism, who had ophthalmopathy and/or upper eyelid retraction, and twelve Hashimoto’s thyroiditis patients, who manifested with only upper eyelid retraction. Approximately 70% of all patients experienced improvement or no change in TED severity while 30% developed increased severity during pregnancy. The authors conclude systemic thyroid autoimmunity and TED are affected differently by pregnancy, though this is limited by small study sample numbers, assessment of only mild-to-moderate TED, and lack of subgroup analysis based on confounding factors such as active or passive smoking and genetic factors [18]. One case reported onset and worsening of Graves’ TED during pregnancy despite treatment initiation. On postpartum day one, the patient experienced complete resolution of eyelid retraction, pain with eye movements, and diplopia. The authors hypothesize TED may worsen during pregnancy secondary to generalized hypervolemia and abruptly improve after significantly decreasing body fluid volume with delivery [19]. More rigorous investigation is warranted to fully explore the effect of pregnancy on TED activity to aid in patient counseling and clinical management.

## 6. Treatment

The manifestations of TED are varied, as seen by the multiple ocular structures affected and different symptoms patients display. While mild TED is generally self-limiting and managed conservatively, severe cases risk vision loss and require more aggressive measures. Additionally, treatment should not be focused solely on the eye, but also take into account the management of a patient’s systemic thyroid disorder. Understanding what treatment regimen to follow requires a team-based approach between the endocrinologist and ophthalmologist, and must be tailored to each individual patient. Several recent studies, and in particular, the updated recommendations and guidelines from the European Thyroid Association (ETA)/European Group in 2021, have helped guide treatment and management for patients globally [20,21,22,23]. 

### 6.1. Management of Hyperthyroidism

Maintaining a euthyroid state decreases the risks of progression of TED. While both medical therapy and subtotal/total thyroidectomy have not been shown to affect TED, the use of radioactive iodine in treating hyperthyroidism has been linked to a worsening eye disease in about 15% of patients within six months of treatment (Figure 3). The risk of TED progression can be decreased by avoiding hypothyroidism after radioactive iodine and by administering a short post-treatment course of oral steroids. Coverage with steroids is not needed with inactive TED and the avoidance of post-treatment hypothyroidism [20].

### 6.2. Smoking

Multiple studies have shown a strong association between smoking and the progression of TED, although the mechanism by which this occurs remains unknown. Smoking may increase the risk of progression by up to 20 times for current smokers in comparison to nonsmokers. Dose-dependent response has also been shown to occur with increased frequency or quantity of smoking. The association of smoking to disease progression is stronger in patients with severe TED. Quitting smoking has also been shown to decrease the risk of TED progression, although this has not been studied extensively. Given the strong association between smoking and the progression of TED, smoking cessation should be strongly encouraged along with referral to a smoking cessation program, if necessary [24].

### 6.3. Mild Thyroid Eye Disease

The majority of patients tend to fall and stay within this category. As the optic nerve is not affected and the majority of symptoms are related to mild ocular surface exposure, treatment is focused on controlling systemic thyroid disorder. Lubricating eye drops during the day and lubricating ointment at bedtime are helpful in treating the symptoms of mild exposure keratopathy. A wedge pillow can be used to raise the head of the bed and decrease the accumulation of periorbital edema. Selenium supplementation has been shown to improve symptoms in mild TED and decrease the risk of progression [25]. It is worth noting that the recommendation for selenium supplementation comes from a European study, where selenium intake is lower than the more selenium-rich diets available in the United States of America [26]. Steroids and radiotherapy should not be used in mild TED as the risks outweigh the benefits [20]. Few patients can receive more advanced treatments if their quality of life is greatly reduced, thus outweighing the risks of treatment [20].

### 6.4. Moderate-to-Severe Thyroid Eye Disease

#### 6.4.1. Acute Disease

The treatment of acute moderate-to-severe TED is rarely surgical, instead focusing on the medical control of the acute inflammatory reaction. Optic neuropathy is not seen in moderate-to-severe TED, but patients are generally symptomatic, and the disorder can be disfiguring. Those who are asymptomatic or unwilling to undergo treatment should be monitored closely. Immunosuppressive treatments are used to control the inflammatory response.

##### Steroids

Steroids are generally used as the first-line treatment and have a large side effect profile. Gastrointestinal acid suppression, vitamin D and calcium supplementation, and bisphosphonates are helpful in mitigating some of the more serious long-term-use side effects of stomach ulcers and osteoporosis. Care should be taken in patients with other comorbidities such as diabetes mellitus and hypertension, and in children and the elderly. Other side effects include insomnia, irritability, weight gain, and acne flares.

Delivery of steroids can be local with periocular injections, oral, or intravenous. Oral steroids are typically dosed at 1 mg/kg, and need to be given for an extended period of time. Early or fast taper may lead to flare of TED. There have been no placebo-controlled trials, but oral steroids have been compared to other treatments with a favorable response in 33–63% of patients. Periocular steroid injections have been used, but outcomes are better with systemic administration [20]. Intravenous steroid infusions have been shown to have better efficacy than oral steroids, with response rates of 80% and 60%, respectively [20,27]. They can be administered as weekly pulse infusions with varying dosages. Doses greater than 500 mg have been reported to have more serious side effects [27], and should be reserved for those with more urgent signs of optic neuropathy. Clinicians should keep in mind the low risk of acute liver damage or failure, arrhythmia, and hypokalemia with a high cumulative dose of steroids (more than eight grams). The decision to use oral or intravenous steroids is dependent upon the individual patient, their comorbidities, and their symptoms. The use of oral or intravenous steroids tends to be equal amongst oculoplastic surgeons in the United States of America, in comparison to the higher rate of intravenous steroid use in Europe and Latin America [28].

##### Radiation

Low-dose external beam radiotherapy can be a useful treatment, although the data are unclear, and the effect is delayed in comparison to steroid therapy [29,30]. Studies showing the utility of radiotherapy are limited. However, it is especially useful in improving extraocular motility issues [27,31]. One commonly used regimen is 20 Gy to each orbit in ten fractionated doses over two weeks. One Gy per week per orbit over 20 weeks has also been used with the same efficacy and better tolerability. Higher doses are not more effective. Other studies have shown even 10 Gy to be as effective as 20 Gy. Simultaneous steroid treatment can reduce the risk of worsened acute symptoms with radiotherapy [20]. Concomitant steroid therapy has also been shown to be more effective than steroid or radiotherapy alone, with intravenous steroids maintaining their superiority to oral steroids [29,30,31,32]. Radiotherapy should be avoided in those under 35 years of age due to the risks of carcinogenesis. Treatment should also not be used in those with diabetes mellitus and hypertension due to the risk of development or worsening of retinopathy. The risk of cataract development is small given the low dose and the low percentage of exposure the lens receives [20,30].

##### Steroid-Sparing Agents

The use of steroid-sparing agents for thyroid eye disease continues to grow. Large, randomized, controlled prospective studies are lacking. These agents have become useful in treating patients unable to tolerate steroids or with contraindications to steroid therapy.

Anticytokine treatments have been used in patients with thyroid eye disease. Etanercept, a TNF receptor blocker, was found to be useful in patients with mild-to-moderate TED [33]. Infliximab, a monoclonal antibody to TNF, was successfully used to treat a patient with disease resistance to oral steroid therapy [33]. Adalimumab, a human monoclonal antibody against TNF-alpha, is the newest of the agents targeting TNF. A retrospective study showed it to be useful in treating patients with active, severe TED resistant to steroid therapy [34].

Intravenous immunoglobulin (IVIG) can also be used to counter the autoantibodies that may be involved in TED. It has been shown to be as effective as oral steroids in the treatment of TED, along with better tolerability [33]. However, studies are lacking, including comparison to intravenous steroids. Additionally, the use of plasma filtration in combination with intravenous methylprednisolone leads to similar improvement as steroid alone. The improvement may be more rapid with the addition of plasma filtration [33]. Similarly, azathioprine has not been shown to improve TED on its own but has shown to be useful when combined with steroids or radiotherapy [29]. Both methotrexate and mycophenolate mofetil have also been shown to be effective when used as monotherapy [33].

Rituximab is a humanized chimeric anti-CD20 monoclonal antibody that depletes B lymphocytes. Multiple case studies have shown promise for the use of rituximab in TED. However, these case series have included multiple confounding factors, including the use of steroids and reporting bias. Rare, serious side effects such as death and optic neuropathy from worsening TED have been reported [35]. A randomized, prospective trial from the Mayo Clinic found no difference in patients with active moderate-to-severe TED to those treated with placebo. About half of the patients in both study groups showed symptomatic and objective improvement, which is consistent with the natural history of TED [35]. A second randomized, prospective trial comparing intravenous methylprednisolone to rituximab did find a statistically significant difference, with 70% response in the steroid group and up to 100% response in the rituximab group. Reactivation of ophthalmopathy was seen in 30% of the steroid-treated patients in comparison to none of the rituximab-treated patients [36]. Given the contradictory results, larger controlled, prospective trials are necessary to further understand the utility of rituximab in the treatment of moderate-to-severe TED.

IL-6 plays a pro-inflammatory role, and is found in high concentrations in orbital adipocytes, fibroblasts, and macrophages of patients with TED. Tocilizumab, a humanized monoclonal antibody to the IL-6 receptor, was shown to be effective in treating patients with active, severe TED who had failed treatment with intravenous steroids and other steroid-sparing agents [33,37]. Adverse effects are generally mild to moderate and transient, including laboratory abnormalities and infections [37].

In 2020, the FDA approved teprotumumab (Tepezza, Horizon Therapeutics, Deerfield, Illinois) for the treatment of thyroid eye disease, which remains the only FDA-approved therapy to date. Teprotumumab is a human monoclonal antibody that blocks the IGF-1 receptor found on T cells that may play a role in the autoimmune process of TED [38]. It is administered as an intravenous infusion every three weeks for eight doses, with an initial dose of 10 mg/kg followed by 20 mg/kg for the remaining doses. Since its advent into the market, several studies have demonstrated its clinical effectiveness in the management of thyroid eye disease [39,40,41]. A multicenter, double-masked, randomized, placebo-controlled trial found that treatment with teprotumumab led to significantly decreased proptosis, Clinical Activity Score (CAS), quality of life scale, and subjective diplopia [39]. A meta-analysis in 2021 reviewed 15 randomized control trials comparing the efficacy of teprotumumab and rituximab, among other treatments, and found that teprotumumab had one of the highest overall response rates [40]. As with any systemic therapy and because IGF-1 is expressed systemically, potential side effects include, but are not limited to: muscle spasms, alopecia, gastrointestinal symptoms, hearing impairment, and hyperglycemia. Possible side effects require close monitoring during treatment, although no standard for monitoring exists to date [38,42].

Although mostly studied in patients with active thyroid eye disease, the benefits of teprotumumab extend to other contexts as well. Recent studies have demonstrated its efficacy in patients with dysthyroid optic neuropathy and a large observational multi-center study demonstrated adequate medical decompression with teprotumumab and objective resolution of dysthyroid optic neuropathy with rapid improvement in vision and reversal of afferent pupillary defects. Teprotumumab has also more recently been studied in the context of chronic, inactive thyroid disease as the IGF-1 receptors persist into the chronic phase. Data from the recent OPTIC-X trial suggests that teprotumumab is efficacious in patients with a disease duration of over one year [43,44]. A recent study also demonstrated reduction in proptosis, improvement in CAS, and improvement in baseline diplopia in a subset of patients with chronic, inactive TED [45]. Other smaller case series have demonstrated similar outcomes in patients with chronic, inactive disease [46,47,48]. However, data on the efficacy of teprotumumab in chronic active and chronic inactive TED patients remains lacking but future plans are in place for further clinical trials.

Several other biologic therapies are currently under study at various stages of clinical trials. Recently, Viridian Therapeutics developed a monoclonal antibody that binds and blocks the IGF-1R signaling pathway, previously also studied in the oncological setting. This new therapy targeting this signaling pathway is named VRDN-001, with alternative subcutaneous versions, VRDN-002 and VRD-003, also in the pipeline. Other IGF-1R inhibitors, namely linsitinib and lonigutamab, are also under study for use in TED. Preliminary clinical data for these new therapies have not yet been released. 

Another potentially promising, subcutaneous therapy option is batoclimab (IMVT-1401), developed by Immunovant, Inc., New York, NY, USA. This new therapy targets the neonatal Fc receptor (FcRn), a receptor responsible for the passive transfer of humoral immunity from mother to neonate. It is proposed that inhibition of this target molecule can reduce immunoglobulin recycling and decrease circulating levels of antibodies, possibly being beneficial in thyroid eye disease [49]. Other similar anti-FcRn drugs, including efgartigimod, rozanolixizumab, nipocalimab, and orlanolimab are currently being studied for other various conditions, but not yet specifically for use in thyroid eye disease. 

#### 6.4.2. Chronic Disease

In patients with stable and controlled systemic thyroid disease, and without signs of change in their moderate-to-severe TED, surgical rehabilitation of the orbit can begin. Patients who are not bothered by their ocular symptoms or appearance should not undergo any orbital surgery. Surgical options include orbital decompression, strabismus surgery, and eyelid procedures. Patients may not need all of these different surgeries. Those requiring more than one type should follow the order as listed, as one surgical type may lead to worsening of another problem.

**Figure 6:**
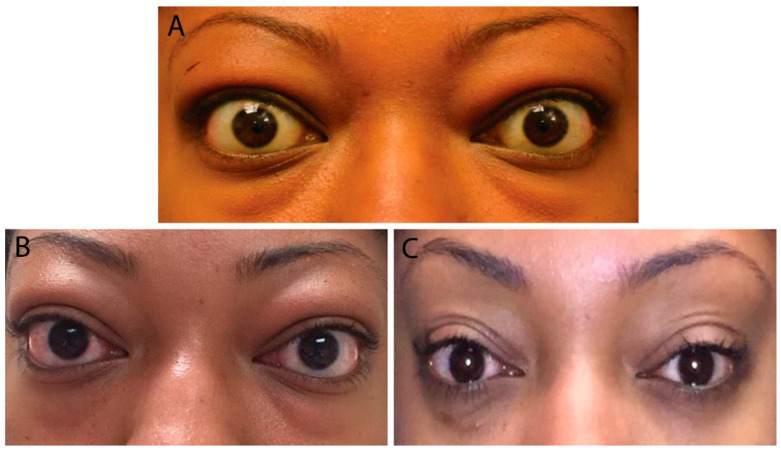
(**A**) External color photograph of acute moderate thyroid eye disease with periorbital edema, upper and lower eyelid retraction, and chemosis (conjunctival swelling). (**B**) Same patient in chronic phase. (**C**) Patient status post-bilateral orbital fat decompression. No further procedures were needed.

Orbital decompression is used to treat exophthalmos. Multiple methods are available without one being found to be superior to another. Patients can undergo a “fat-only” decompression to reduce the orbital volume without affecting the orbital architecture (Figure 6) [50]. This is more useful in patients who do not have excessive muscle enlargement causing crowding of the optic nerve at the orbital apex, as this area is not effectively decompressed with only fat removal. Patients with especially large muscles encroaching on the optic nerve are better served by a “bony” decompression where the orbital walls are partially removed to create room for the orbital contents. Risks include permanent vision loss, double vision, and the need for further procedures. The risk of double vision may be reduced by “balanced” medial and lateral wall decompressions, excluding the orbital floor [51,52]. Maintenance of the “orbital strut”, the inferomedial bony transition between the medial orbital wall and floor, has been advocated in preventing postoperative diplopia [41]. Patients should understand the mentioned risks along with many others prior to undertaking a surgical procedure such as orbital decompression. 

**Figure 7:**
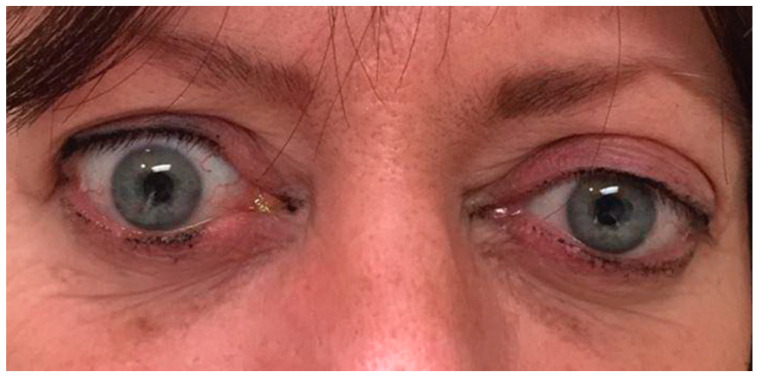
External color photograph depicting a patient with strabismus. The right eye is slightly elevated and abducted in comparison to the left eye, as noted by the position of the corneal light reflex. A slight left head tilt is also taken to help combat the double vision. Right upper eyelid retraction is also noted.

Patients with double vision can be treated with prism glasses or surgery. Prism glasses shift images to allow fusion with both eyes. They tend to be more useful in patients with smaller amounts of strabismus (Figure 7). Large amounts of strabismus are difficult to treat with prisms due to the optical difficulties of large prisms. The goal of strabismus surgery in TED is to achieve binocular fusion in primary gaze (looking straight ahead) and in downgaze. Given the large scarred muscles in TED with different muscles being affected by different amounts, the correction of double vision in all fields of gaze is often not possible. 

### 6.5. Vision-Threatening Thyroid Eye Disease

#### 6.5.1. Optic Neuropathy

Optic neuropathy is usually due to compression of the optic nerve at the orbital apex. The optic nerve does have some inherent slack, allowing for proptosis with the increase in fibroadipose tissue and extraocular muscle size. However, there is limited space at the orbital apex for the muscles and optic nerve. Enlargement of the extraocular muscles with compression of the optic nerve or excessive stretching of the nerve from proptosis causes vision loss that can lead to permanent blindness if left unrecognized. It is often difficult to distinguish the symptoms of optic neuropathy from more benign causes such as dry eye. As such, regular visits with an ophthalmologist are of utmost importance for thyroid patients.

Signs of optic neuropathy include blurred vision, decreased color vision or saturation, dimming of vision, or other vision loss. These symptoms should alert the clinician to arrange an urgent evaluation with the patient’s ophthalmologist. Other concerning signs include subluxation of the globe (eye popping out past the eyelids) or acute increase in orbital edema and proptosis. It is important to note that globe subluxation should be immediately treated by the provider or patient by pushing the eye back into the orbit with a wet gauze, glove, or clean hand as this situation can lead to rapid vision loss.

The treatment of optic neuropathy is with steroids or orbital decompression, either alone or in combination. Given the urgency in treatment, high-dose intravenous (IV) steroids (500–1000 mg methylprednisolone daily, as a single dose or divided doses) are given with a taper to oral steroids as clinically indicated. Milder cases of optic neuropathy can be treated with pulse IV steroids. Cases of optic neuropathy that are not improving with IV steroids can be treated with orbital decompression. Treatment with immediate decompression has not been shown to be superior to IV steroids, and should be reserved for cases when it is absolutely necessary [20,53]. It is important to note that orbital radiotherapy should not be used for the treatment of optic neuropathy due to the delayed effect, unless it is used as an adjunct to other treatments. Steroid-sparing agents can be used when intravenous steroids do not improve the optic neuropathy, side effects are intolerable, or treatment is contraindicated, and surgical intervention is not yet required.

#### 6.5.2. Severe Corneal Exposure

Eyelid retraction and proptosis, alone or in combination, cause corneal exposure and drying. Chronic dryness leads to corneal thinning, breakdown, and eventual perforation. Lack of a Bell’s phenomenon can lead to further damage. These patients must lubricate frequently, preferably with ointment throughout the day. Dry eye goggles or moisture chambers may also be useful. Upper eyelid retraction can also be temporarily improved with botulinum toxin injected into the upper eyelid to reduce lid retraction or provide a protective ptosis, although onset is delayed by a few days, and treatment is difficult to titrate [54]. Steroid eye drops should be avoided as they inhibit corneal healing. Those with excessive exposure may require closing the eyelids laterally with a suture, either in a temporary or “permanent” fashion. Orbital decompression followed by possible eyelid retraction repair may be needed to bring the eye and eyelids into proper position. Patients who do suffer a corneal perforation may require topical antibiotics along with corneal gluing and a bandage contact lens, or a surgical correction if necessary.

### 6.6. Special Considerations

#### 6.6.1. Pediatric Thyroid Eye Disease Treatment

Although rare, clinicians should be aware of treating TED in children. Focus is on systemic treatment just as in adults. TED tends to be mild and resolves with systemic control. Exposure to smoking and second-hand smoke must be limited. As in adults, lubricating eye drops and ointment are recommended for mild TED. Steroids should be avoided given the risk of growth suppression, unless optic neuropathy is noted and requires treatment. A low oral dose of 5–20 mg for 4–6 weeks with subsequent taper has been advocated [1]. Intravenous steroids may be used if necessary. The effects of immunomodulatory medications in children remain unclear. Radiotherapy cannot be used in children, and should be avoided in young adults under 35 years of age. Surgical options are available as in adults, but should be limited in use unless necessary, especially in acute disease [20].

#### 6.6.2. Pregnancy

Although this presents a rare situation with the worsening of thyroid eye disease to involve optic neuropathy during pregnancy, the clinician should be prepared to treat this situation. Although thyroid eye disease may begin to worsen in the first trimester, it tends to improve during the second trimester through a period of relative immunosuppression, reactivating again after delivery [51]. However, some patients may continue to worsen and may progress to optic neuropathy. These patients should be treated with intravenous high-dose steroids in closed consultation with the obstetrician. The risk of fetal teratogenicity of steroids is thought to be very low [55]. If steroids do not improve the optic neuropathy, orbital decompression should be performed. Although the second trimester is the preferred time to operate during pregnancy, an emergent situation such as optic neuropathy requires surgical treatment regardless of the trimester [56].

## 7. Conclusions

TED remains an enigmatic disorder of the orbit despite decades of fertile research into elements ranging from its molecular pathogenesis to its clinical significance on patients’ quality of life. What we do know is that there is potential for devastating visual, functional, and cosmetic consequences. It is imperative to understand disease recognition, appropriate work up, and timely, appropriate management. Long-term, those who study TED must strive for more rigorous investigation into the aspects of TED that will minimize the potential for irreparable damage and enable the greatest patient care.

## Nomenclature

CT	Computed tomography
EUGOGO	European Group on Graves’ Orbitopathy
IGF-1	Insulin-like growth factor-1
IL	Interleukin
IV	Intravenous
IVIG	Intravenous immunoglobulin
MRI	Magnetic resonance imaging
NOSPECS	N: no signs or symptoms; O: only signs; S: soft-tissue involvement; P: proptosis; E: extraocular muscle involvement; C: corneal involvement; S: sight loss due to optic nerve compression
TBII	Thyroid-binding inhibitory immunoglobulin
TED	Thyroid eye disease
TPO	Thyroid-peroxidase
TSAb	Thyrotropin receptor antibody
TSH	Thyroid-stimulating hormone
TSHR	Thyroid-stimulating hormone receptor
TSI	Thyroid-stimulating immunoglobulin
VISION	V: vision, optic neuropathy; I: inflammation, congestion; S: strabismus, motility restriction; A: appearance, exposure
